# Effect of a Synbiotic Combination of 2′-Fucosyllactose and *Lactiplantibacillus plantarum Hi188* on Skeletal Growth and Gut Microbial Metabolism in Growing Mice

**DOI:** 10.3390/nu18071123

**Published:** 2026-03-31

**Authors:** Jian Kuang, Yang Li, Linjun Wu, Xiangyu Bian, Jianqiang Li, Fangshu Shi, Xiaoqiong Li, Xin Wang, Jinzhu Pang, Jinjun Li

**Affiliations:** 1 State Key Laboratory for Quality and Safety of Agro-Products, Zhejiang Academy of Agricultural Sciences, Hangzhou 310021, China; jian_kuang95@yeah.net (J.K.);; 2 Key Laboratory of Postharvest Preservation and Processing of Vegetables (Co-Construction by Ministry and Province), Ministry of Agriculture and Rural Affairs, Zhejiang Key Laboratory of Intelligent Food Logistic and Processing, Institute of Food Science, Zhejiang Academy of Agricultural Sciences, Hangzhou 310021, China; 3 Mengniu Institute of Nutrition Science, Global R&D Innovation Center, Inner Mangolia Mengniu Dairy (Group) Co., Ltd., Beijing 101107, China

**Keywords:** 2′-FL, bone density, SCFAs, bone growth, synbiotics, gut microbiota, micro-CT

## Abstract

**Background/Objectives:** Early-life skeletal growth is critical for achieving optimal peak bone mass. This study aimed to investigate whether synbiotic supplementation with 2′-fucosyllactose (2′-FL) and *Lactiplantibacillus plantarum Hi188* (*Hi188*) influences bone development in growing mice and its potential association with gut microbial modulation. **Methods:** Three-week-old BALB/c mice were orally supplemented with 2′-FL, *Hi188*, or their combinations at low and medium doses for 7 weeks. Bone length, microarchitecture and mechanical properties were analyzed. Serum bone turnover markers and osteogenic gene expression were analyzed by ELISA and qRT-PCR, respectively. Gut microbiota composition, and short-chain fatty acid (SCFA) production were analyzed by 16S-rRNA sequencing and GC-FID, respectively. Correlation associations among microbial taxa, SCFAs, and skeletal parameters were also assessed. **Results:** Medium-dose synbiotic supplementation significantly increased tibial and spine length without altering body weight or intestinal histology. Trabecular bone density and volume fraction were elevated, accompanied by reduced trabecular separation and improved mechanical strength. Serum BALP levels were increased and TRACP-5b levels were reduced, together with upregulation of osteogenesis- and matrix-related genes. Synbiotic treatment also modulated gut microbial composition, enriched SCFA-associated taxa, and significantly enhanced total and individual SCFA levels. Correlation analyses revealed selective associations among specific microbial taxa, SCFAs, and skeletal structural and molecular parameters. **Conclusions:** Medium doses of synbiotic supplementation were associated with improved skeletal growth and bone quality during development, alongside coordinated modulation of bone remodeling and gut microbial metabolic activity. These findings suggest its potential as a functional nutritional strategy for supporting early bone health.

## 1. Introduction

Linear growth and bone mass accrual during childhood and adolescence are critical determinants of peak bone mass and long-term skeletal health [1]. During this period, bone modeling and remodeling are highly dynamic and sensitive to multiple regulatory factors, including nutritional supply, endocrine signaling, and immune modulation [2]. In recent years, the gut–bone axis has emerged as an important framework for understanding how dietary factors may influence bone growth [3,4]. Through microbial fermentation, immune regulation, and nutrient absorption, the gut microbiota acts as a key mediator relevant to bone formation and resorption [5]. However, although microbiota-targeted nutritional interventions have attracted increasing interest, the specific combinations and mechanisms most relevant to skeletal growth during development remain incompletely defined.

2′-fucosyllactose (2′-FL), the most abundant fucosylated structure among human milk oligosaccharides (HMOs), has received substantial attention because of its ability to shape the intestinal microbial environment, support barrier function, and modulate host immunity [6,7,8]. Recent animal studies have begun to uncover a direct link between 2′-FL supplementation and bone metabolism. In aging-associated bone loss models, 2′-FL was reported to be associated with bone mineral density and trabecular microarchitecture by remodeling the gut microbial community and attenuating innate immune activation, highlighting its potential role in preventing osteoporosis [9]. Beyond 2′-FL, HMOs in a broader context have been implicated in shaping bone biology. In malnourished or germ-free juvenile mouse models, supplementation with sialylated HMOs significantly increased trabecular bone volume fraction and cortical thickness, while suppressing osteoclastogenesis without impairing osteoblastic activity [10]. Although direct evidence linking 2′-FL to longitudinal skeletal development during growth remains limited, converging data from these studies support a mechanistic framework wherein fucosylated and sialylated HMOs may influence bone homeostasis via gut microbiota-derived metabolites, immune–bone coupling, and osteoclast inhibition [11]. These insights provide a scientific basis for exploring 2′-FL as a potential nutritional strategy to promote bone mass accrual and optimize skeletal health during growth.

*Lactiplantibacillus plantarum*, is a widely studied probiotic species with documented effects on host growth, metabolism, immune regulation, and intestinal barrier function [12]. In juvenile murine models, certain *Lactobacillus* strains have been reported to maintain body weight and promote longitudinal growth under conditions of nutritional restriction, possibly through interactions with the somatotropic axis, including IGF-1-related signaling [13]. In addition, the growth-promoting effects of *L. plantarum* are strain-dependent, indicating that specific isolates exert distinct influences on host development. Multiple *L. plantarum* strains have been shown to ameliorate bone loss in osteoporosis models induced by ovariectomy or glucocorticoid exposure [14,15,16]. Notably, these probiotic effects are strain-dependent, indicating that individual isolates may differ substantially in their biological activities. *Lactiplantibacillus plantarum Hi 188 (Hi188)* is a newly isolated strain with no prior reports regarding its effects on growth or skeletal development, providing a unique opportunity to explore strain-specific probiotic–bone interactions during developmental stages.

Based on the above considerations, we selected 2′-FL and *Hi188* as a biologically motivated candidate synbiotic combination for evaluation in a juvenile mouse model. Importantly, this pairing was not based on prior direct evidence that 2′-FL is a preferential growth substrate for *Hi188*. Rather, the rationale was that 2′-FL and *Hi188* may each modulate the intestinal ecosystem through complementary routes, thereby potentially producing greater benefits in combination than when administered alone. In this context, the present study was designed to test the biological effectiveness of this combined intervention, rather than to establish strain-specific substrate utilization.

Short-chain fatty acids (SCFAs), the principal fermentation products of dietary carbohydrates by gut microbiota, exert dual beneficial effects on bone health [3,5]. In particular, butyrate promotes bone formation by activating the Treg–CD8^+^ T cell axis, which upregulates Wnt10b signaling in osteoblast precursors [5,17,18]. Consistent with this, recent systematic reviews highlight SCFAs as central mediators of the gut–bone axis, maintaining bone homeostasis even under osteoporotic conditions [19,20]. Nevertheless, whether microbiota-targeted interventions improve skeletal development through SCFA-related pathways in growing animals remains insufficiently resolved. Therefore, SCFAs were considered in the present study as potential microbiota-associated mediators of interest, rather than as predefined causal drivers.

Although both 2′-FL and *Lactiplantibacillus plantarum* have been independently reported to influence the microbiota–immune–bone axis, it remains unclear whether their combined supplementation affects longitudinal growth and bone mass accrual during development. In addition, the microbiota- and metabolite-associated mechanisms potentially involved in these effects remain insufficiently defined. We hypothesized that combined supplementation with 2′-FL and *Hi188* might improve growth-related and bone-related outcomes during development, potentially in association with gut microbiota remodeling and altered fecal SCFA profiles. To test this hypothesis, we comprehensively evaluated morphometric parameters, biochemical markers, bone microarchitecture by micro-CT, and microbiome outcomes in a murine growth model, using NC mice as the control group. This research is expected to fill the knowledge gap in this field and to inform the development of targeted nutritional strategies for optimizing skeletal health during growth.

## 2. Materials and Methods

### 2.1. Preparation and Administration of Lactiplantibacillus plantarum Hi188 and 2′-Fucosyllactose

The large anaerobic workstation (ELECTROTEK, Aberdeen, UK) was used to cultivate the probiotic *Lactiplantibacillus plantarum Hi 188 (Hi188)*, which was supplied by China Mengniu Dairy Company Limited (Inner Mangolia Mengniu Dairy Co., Ltd., Beijing, China), in De Man, Rogosa and Sharpe (MRS) broth for 24 h at 37 °C under anaerobic conditions. After that, cultured bacteria were harvested by centrifugation (8000× *g*, 4 °C, 5 min), washed three times with sterile phosphate-buffered saline (PBS, Solarbio, Beijing, China), and resuspended in sterile PBS for oral gavage. The bacterial suspension was freshly prepared each day, and its concentration was adjusted by the McFarland turbidity method. Before the animal study, this method was validated for *Hi188* by serial dilution and plate counting to establish the relationship between turbidity and viable CFU. The final gavage dose was calculated according to body weight to achieve the intended CFU/kg/day.

2′-fucosyllactose was also supplied by China Mengniu Dairy Company Limited (Inner Mangolia Mengniu Dairy Co., Ltd., Beijing, China) and was dissolved in sterile PBS and freshly prepared before administration. The concentration of the 2′-FL solution was adjusted according to the body weight of the mice to ensure accurate delivery of the designated dose.

### 2.2. Animals, Diets and Experimental Design

The animal study was reviewed and approved by the Ethics Committee of the Zhejiang Academy of Agricultural Sciences (approval No: 25ZALAS10). Male BALB/c mice, weighing 18–20 g and aged 3 weeks, were purchased from GemPharmatech Co., Ltd. (Nanjing, China) and housed at the Zhejiang Academy of Agricultural Sciences’ Animal Experimental Center. They were kept in conditions that were suitable for their specific pathogen-free (SPF) status (20–22 °C, 40–60% humidity, and a 12 h light/dark cycle). The mice were acclimated to various feeding schedules following one week of unrestricted access to food and water. A total of 40 mice were randomly divided into five groups (8 mice per group): the control group (NC), the probiotic group (*Hi188*), the 2′-FL group (2′-FL), and two synbiotic groups receiving either a low dose (Syn-L) or a medium dose (Syn-M). A schematic overview of the experimental design is provided in Supplementary Figure S4. The *Hi188* and 2′-FL groups served as single-agent reference groups, whereas Syn-L and Syn-M were included as reduced-dose combination interventions. Mice were group-housed with four animals per cage, and each experimental group was distributed across two cages. Throughout the experiment, mice had free access to an irradiated laboratory rat and mouse growth and breeding diet (Jiangsu Xietong Pharmaceutical Bio-engineering Co., Ltd., Nanjing, China) and water. According to the manufacturer’s specifications, the diet contained 13.7 g/kg calcium, 9.2 g/kg phosphorus, and 1900 IU/kg vitamin D, based on 90% dry matter. During the 7-week intervention period, mice in the control groups were fed a regular diet and gavaged with 200 μL of sterile PBS. The probiotic group received *Hi188* at a dose of 1 × 10^9^ CFU/kg/day. Mice in the 2′-FL group were given 2′-FL at a dose of 0.4 g/kg/day. The synbiotic group was split into two dose subgroups: 2.5 × 10^8^ CFU/kg/day + 0.1 g/kg/day was given to the low-dose group, and 5 × 10^8^ CFU/kg/day + 0.2 g/kg/day was given to the medium-dose group. For 7 weeks, the medications were administered, with regular monitoring of the animals’ body weights.

At the end of the experimental period, mice were deeply anesthetized with sodium pentobarbital, and blood samples were obtained from the retro-orbital sinus. Mice were subsequently euthanized by cervical dislocation in accordance with institutional animal care guidelines. Serum was centrifuged at 4000 rpm for 10 min and stored at −80 °C for further analyses. The liver, heart, spleen, lung, and kidney were excised and analyzed, and the lengths of the spine and tibia were recorded. Femurs were collected and soaked in 4% paraformaldehyde for micro-CT and three-point bending analyses. A 4–5 mm segment of the distal colon was collected and fixed in 4% paraformaldehyde for hematoxylin and eosin (H&E) staining. Distal tibial segments were collected and stored at −80 °C for quantitative real-time PCR (qPCR) analysis. Intestinal contents were collected under sterile conditions and stored at −80 °C for 16S rRNA gene sequencing, while fresh fecal samples were collected before euthanasia and directly stored at −80 °C for short-chain fatty acid (SCFA) analysis.

### 2.3. Measurement of Tibial and Spine Length

Spine length was measured as the distance of the trunk spine, and the tibial length was measured from the proximal end to the distal end of the tibia. (*n* = 40, 8 mice per group) using a INOX IP54 vernier caliper (Micro Precision Calibration Inc., Grass Valley, CA, USA), with a resolution of 0.01 mm.

### 2.4. Measurement of Serum Bone Turnover Markers

The mice were kept in a fasting and water-free state for 12 h. Fresh orbital blood was then collected, centrifuged for 10 min at 4 °C at 4000 rpm, and stored for long-term in a refrigerator at −80 °C. Concentrations of bone formation and resorption markers were measured as follows: bone-specific alkaline phosphatase (BALP), osteocalcin (OC), tartrate-resistant acid phosphatase 5b (TRACP-5b), and *N*-terminal telopeptide of type I collagen (NTX). All assays were performed using commercially available ELISA kits according to the manufacturers’ instructions (Beijing Biotopped Science Technology Co., Ltd., Beijing, China; catalog numbers: BALP TOPEL30257, OC TOPEL30258, TRACP-5b TOPEL02080, NTX TOPEL303092). Serum samples and standards were run in duplicate. Briefly, thawed serum aliquots were equilibrated to room temperature, mixed gently, and 50–100 µL was added per well (volume per manufacturer’s instructions). Plates were incubated and washed according to the kit protocol. Optical density was read using a microplate reader at the wavelength recommended by the manufacturer. Concentrations were calculated from standard curves generated by four- or five-parameter logistic regression.

### 2.5. Micro-CT Analysis

The left femurs were soaked in 4% paraformaldehyde (Servicebio, Wuhan, China) over 24 h and then analyzed immediately using a micro-CT system (VNC-102, Pinglin Medical Technology Co., Ltd., Zaozhuang, China) using 90 kV voltage, 0.09 mA current, and 10 μm resolution in the distal growth plate of femurs. After the scan was completed, the 3D images were reconstructed. Cruiser software (2.013.2) was used to adjust the direction and other parameters of the scanned sample images to ensure that all samples were processed under the same conditions to generate VOI images. Subsequently, the original images were reconstructed using the 3D reconstruction software Recon (2.013.2). Then, the target region ROI was analyzed using the data analysis software Avatar (2.012.9). All samples were selected from the same area for analysis to obtain the required parameter values. The single VOI image of each sample was imported to select 100–200 layers below the femoral growth plate to obtain bone morphometric parameters, including bone mineral density (BMD), bone volume fraction (BV/TV), trabecular thickness (Tb.Th), trabecular separation (Tb.Sp) and trabecular number (Tb.N). BV/TV is the ratio of the total volume of the voxels representing bone structures in the ROI to the total volume of all voxels in the region. Tb.Th is the average thickness of the trabecular bone. Tb.N is the number of intersections between bone tissue and non-bone tissue in a given length of bone. Tb.Sp is the average width of the pulp cavity between the trabeculae, indicating the porosity of the trabecular bone.

### 2.6. Bone Biomechanical Testing

The left femur samples were tested using a three-point bending test on an Instron E10000 Universal Material Mechanical Testing Machine (Instron E10000, Instron Company, Boston, MA, USA). Each femur was positioned horizontally on two lower supports with the broad side facing upward. The support span was fixed at 6 mm for all samples and kept constant across all groups to ensure uniform testing conditions. The workstation was operated to make the probe of the tester slowly drop; the loading speed of the probe was 3 mm/min and continued until the femur broke. The original data and compression curve were obtained through calculation. The bone mechanical characteristics were analyzed, including bending stress, flexural stiffness, and maximum load.

### 2.7. Histological Analysis of the Distal Colon

A 4–5 mm segment of the distal colon was collected and fixed in 4% paraformaldehyde to preserve tissue architecture and antigenicity. Subsequently, the fixed sections were processed for routine hematoxylin and eosin (H&E) staining to evaluate histopathological changes. The detailed staining protocol has been described previously [21].

### 2.8. RNA Preparation and Gene Expression Analysis by Real-Time qPCR Analysis

Distal tibial tissue was collected immediately after sacrifice. The distal tibia segment containing the growth plate and adjacent cartilage (without separation/removal of cartilage or growth plate) was excised, snap-frozen in liquid nitrogen, and stored at −80 °C until analysis. Total RNA was extracted from the frozen tissue using the Trizol reagent (TransGen Biotech, Beijing, China) and reverse-transcribed using TransScript All-in-One First-Strand cDNA Synthesis Kit (TransGen Biotech, Beijing, China). A reverse-transcription polymerase chain reaction (RT-PCR) was conducted with SYBR Green on the Bio-Rad CFX96 Real-Time PCR system (Bio-Rad Laboratories, Hercules, CA, USA). Gene expression in each sample was normalized using glyceraldehyde 3-phosphate dehydrogenase (Gapdh). Relative quantification was performed using the 2-ΔΔCt method. The primers used in this study are listed in Table 1.

### 2.9. SCFA Detection

The content of SCFAs, including acetic acid, propionic acid, butyric acid, isobutyric acid, valeric acid, and isovaleric acid, in freshly voided fecal samples was determined by gas chromatography–mass spectrometry (Agilent Technologies, Santa Clara, CA, USA) using the same method as described in the previous experiment [22,23] with minor modifications. Briefly, fecal samples were homogenized to prepare a 10% (*w*/*v*) suspension, and the supernatant was collected after centrifugation. For SCFA extraction, 500 μL of the sample extract was mixed with 100 μL of a crotonic-acid-containing metaphosphoric acid solution as the internal standard and acidifying reagent, and then frozen, thawed, centrifuged, and filtered through a 0.22 μm membrane before analysis. SCFAs, including acetic acid, propionic acid, isobutyric acid, butyric acid, isovaleric acid, and valeric acid, were determined using gas chromatography with flame ionization detection (GC-FID) equipped with an FFAP capillary column (30 m × 0.25 mm × 0.25 μm). The oven temperature was initially set at 75 °C, increased to 180 °C at 20 °C/min and held for 1 min, then raised to 220 °C at 50 °C/min and held for 3 min. The injector and detector temperatures were both set at 250 °C. Nitrogen was used as the carrier gas at a flow rate of 2.5 mL/min, with an injection volume of 1.0 μL and a split ratio of 10:1. SCFAs were identified by the comparison of retention times with authentic standards and quantified using the internal standard method based on calibration curves.

### 2.10. 16S rRNA Sequencing

The results of 16s rRNA amplicon sequencing were obtained and analyzed by LC-Bio Technologies Co., Ltd. (Hangzhou, China). The V3–V4 region of the bacterial 16S rRNA gene was amplified by PCR using the universal primer pair 341F/805R (341F: 5′-CCTACGGGNGGCWGCAG-3′; 805R: 5′-GACTACHVGGGTATCTAATCC-3′). The raw sequencing data were initially processed by merging paired-end reads based on their overlapping regions, followed by filtering low-quality sequences and chimeras to obtain high-quality clean data. Subsequently, the clean data were processed through the QIIME2 pipeline, with sequence denoising performed using the DADA2 plugin to remove potential PCR and sequencing errors, ultimately generating amplicon sequence variants (ASVs) and their corresponding abundance table. This data was then used for downstream analyses, including the assessment of alpha diversity (Chao1, pielou_e, Simpson and Shannon index) and beta diversity (visualized via PCA and non-metric multidimensional scales). Taxonomic classification was conducted using the NCBI and SILVA reference databases to analyze microbial composition at the phylum and genus levels. Linear discriminant analysis effect size (LEfSe, LDA ≥ 2.0, *p* value < 0.05) was employed to identify differentially abundant taxa between groups, and functional pathway prediction was performed using PICRUSt2 based on the 16S rRNA profiles.

### 2.11. Statistical Analysis

All statistical analyses were performed using SPSS 26.0. Data are shown as mean ± SEM. Normality was assessed using the Shapiro–Wilk test and homogeneity of variance was assessed using Levene’s test before selecting parametric or non-parametric tests. For normally distributed data, differences among multiple groups were using one-way ANOVA followed by Tukey’s post hoc test. For non-normally distributed data, differences among multiple groups were analyzed using the Kruskal–Wallis test followed by Dunn’s post hoc test, and the Mann–Whitney U test was used for comparisons between two groups. Body weight over time was analyzed using repeated-measures ANOVA. Different letters indicate statistically significant differences among groups (*p* < 0.05). Spearman correlation analysis was performed to explore the potential associations between genus-level microbial taxa and host phenotypes. *p* values from the Spearman correlation analysis were adjusted using the Benjamini–Hochberg false discovery rate (BH-FDR) procedure in Microsoft Excel. Correlations with FDR-adjusted *p* values < 0.05 were considered statistically significant.

## 3. Results

### 3.1. The Feeding of the Medium-Dose Group Enhanced the Length of Femur and Spine

**Figure 1:**
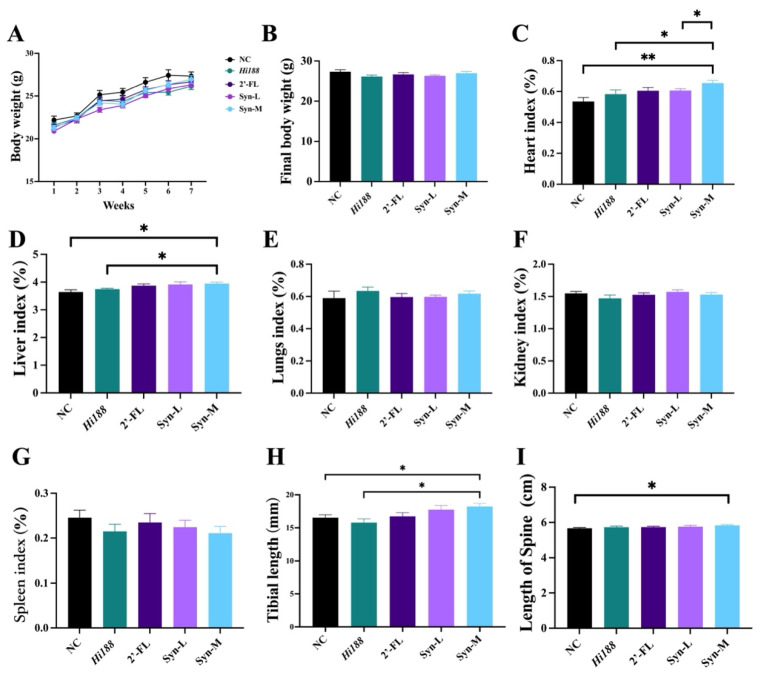
Physiological indices of mice. (**A**) Body weight over time, (**B**) changes in final body weight (g) in mice. The index of heart (**C**), liver (**D**), lungs (**E**), kidney (**F**), spleen (**G**). (**H**) Tibial length. (**I**) Length of spine. Data are presented as mean ± SEM (*n* = 8). Statistical significance from (**B**–**I**) was assessed using parametric or non-parametric tests as appropriate, as described in the Section 2.11. Different asterisks indicate statistically significant differences among groups (*, *p* < 0.05; **, *p* < 0.01).

To investigate the bone-promoting effects of the 2′-FL & *Hi188* complex, we employed 3-week-old mice and administered *Hi188*, 2′-FL, or gradient doses of the synbiotic complex via oral gavage. Body weight, a key indicator of growth and development, was monitored weekly. At baseline, the NC group exhibited a slightly higher body weight than other groups; however, the differences were not statistically significant. Over the course of feeding, body weight increased progressively across all groups, with no significant intergroup variations (Figure 1A,B). We next assessed the organ index, defined as the ratio of organ weight to body weight (Figure 1C–G). No significant differences were observed among groups for the spleen, lung, and kidney indices (Figure 1E–G). In contrast, the liver index was significantly elevated in the Syn-M compared with the NC and *Hi188* groups (*p* < 0.05, Figure 1D). Similarly, the heart index was significantly higher in Syn-M than in NC (*p* < 0.01), and was also elevated relative to the Syn-L group (*p* < 0.05, Figure 1C). To further evaluate skeletal growth, tibial length and spine length were measured (Figure 1H,I). The tibial length in the Syn-M was markedly increased relative to both the NC and *Hi188* groups (*p* < 0.05, Figure 1H). Spine length was also greater in Syn-M compared with the NC group (*p* < 0.05, Figure 1I).

### 3.2. The Feeding of the Medium-Dose Group Enhanced the Bone Density of the Mice

**Figure 2:**
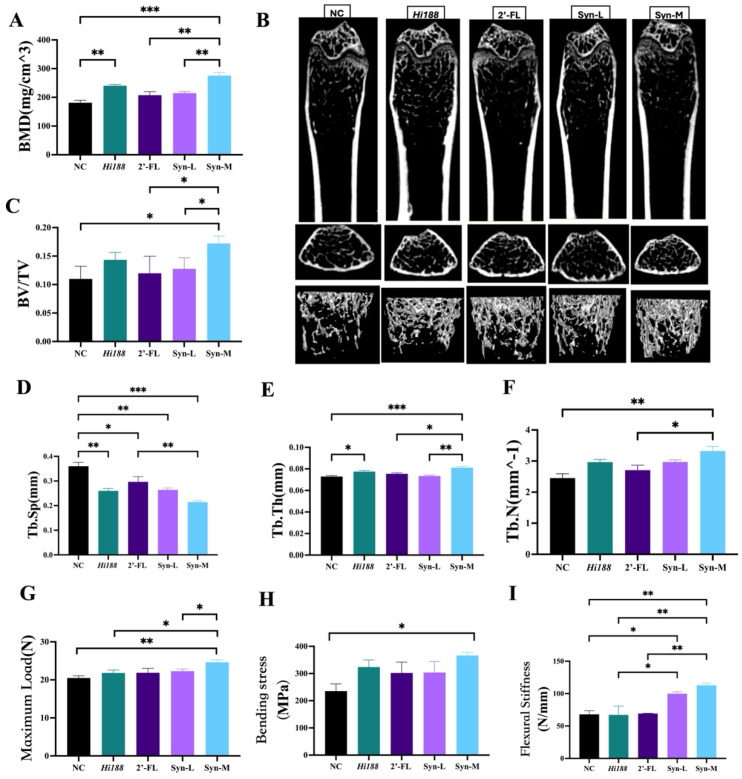
The effect of CBP on bone quantitative indices. (**A**) The BMD difference between the groups. (**B**) Three-dimensional microstructure of trabecular bone in the femur. Bone trabecular indicators including BV/TV (**C**), Tb.Sp (**D**), Tb.Th (**E**) and Tb.N (**F**). The mechanical properties of bones, including the maximum load (**G**), stress (**H**), and stiffness (**I**). The data are presented as mean ± SEM. Statistical significance was assessed using one-way analysis of variance (ANOVA) followed by Tukey’s post hoc test. Different asterisks indicate statistically significant differences among groups (*, *p* < 0.05; **, *p* < 0.01; ***, *p* < 0.001).

To further characterize the structural and functional quality of bone tissue, micro-computed tomography (micro-CT) and biomechanical analyses were subsequently performed. Micro-CT (Figure 2A–F) was used to assess bone volume fraction (BV/TV), bone mineral density (BMD), trabecular number (Tb.N), trabecular thickness (Tb.Th), and trabecular separation (Tb.Sp), which together provide quantitative information on bone mass and trabecular microarchitecture. In addition, biomechanical testing (Figure 2G–I) was conducted to determine bone strength and stiffness, offering functional evidence to complement the structural findings. As shown in Figure 2A,C–F, the Syn-M markedly enhanced bone mineral density (BMD), bone volume fraction (BV/TV), trabecular number (Tb.N), and trabecular thickness (Tb.Th), while significantly reducing trabecular separation (Tb.Sp), compared with the NC group (*p* < 0.05). A similar trend was observed when compared with the 2′-FL group. Compared with the Syn-L group, the Syn-M treatment significantly increased BMD, BV/TV, and Tb.Th, whereas Tb.N showed an increasing trend without reaching statistical significance. Relative to the *Hi188* group, Syn-M exhibited consistent directional improvement across multiple microarchitectural parameters; however, these differences were not uniformly statistically significant. The three-dimensional reconstruction of trabecular bone microarchitecture (Figure 2B) further illustrated these differences. Relative to the NC group, mice receiving *Hi188*, 2′-FL, or synbiotics of varying doses exhibited reduced intra-trabecular spacing, with the Syn-M showing the most pronounced effects. Quantitatively, the Syn-M treatment increased BV/TV by 56.60%, BMD by 52.10%, Tb.Th by 11.30%, and Tb.N by 35.63%, while decreasing Tb.Sp by 40.48% compared with the NC group.

Biomechanical properties further supported these structural findings (Figure 2G–I). The Syn-M exhibited significantly improved bending stiffness, maximum load, and flexural stiffness relative to the NC group (*p* < 0.05), indicating enhanced bone strength under treatment. Moreover, the Syn-M demonstrated a significantly greater maximum load than both the Syn-L and *Hi188* groups (*p* < 0.05). For flexural stiffness, both the Syn-M and Syn-L groups showed significant increases compared with the NC and *Hi188* groups, although no significant difference was observed between the two doses.

### 3.3. Effects of the Intervention on Colonic Morphology

**Figure 3:**
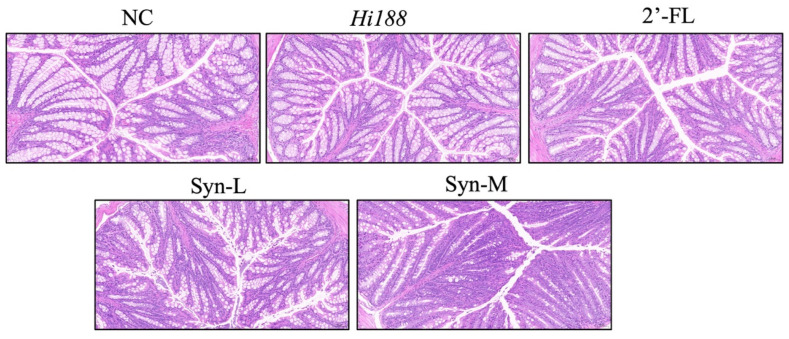
The effects of feeding in different groups on colonic H&E staining. Scale bar = 50 μm.

Since bone growth is closely influenced by intestinal health and nutrient absorption, we next examined colonic morphology using hematoxylin and eosin (H&E) staining. The colon contributes to the absorption of key bone-supporting nutrients—such as calcium and phosphate—and acts as the main fermentation site for microbial metabolism of dietary fibers into short-chain fatty acids (SCFAs) [20,23,24], which enhance mineral absorption and regulate bone remodeling. Impaired colonic structure, such as mucosal damage or crypt distortion, can reduce nutrient uptake and alter gut-derived signaling pathways, thereby indirectly affecting bone formation. As shown in Figure 3, no overt histopathological abnormalities were observed in the colonic tissues of mice treated with *Hi188*, 2′-FL, or their synbiotic combinations. The overall mucosal organization and crypt architecture appeared comparable among all groups, with an intact epithelial structure and no evident signs of mucosal disruption or inflammatory cell infiltration. These results indicate that the applied interventions did not induce noticeable alterations in colonic morphology under the present experimental conditions.

### 3.4. Synbiotics Primarily Promote Bone Formation

**Figure 4:**
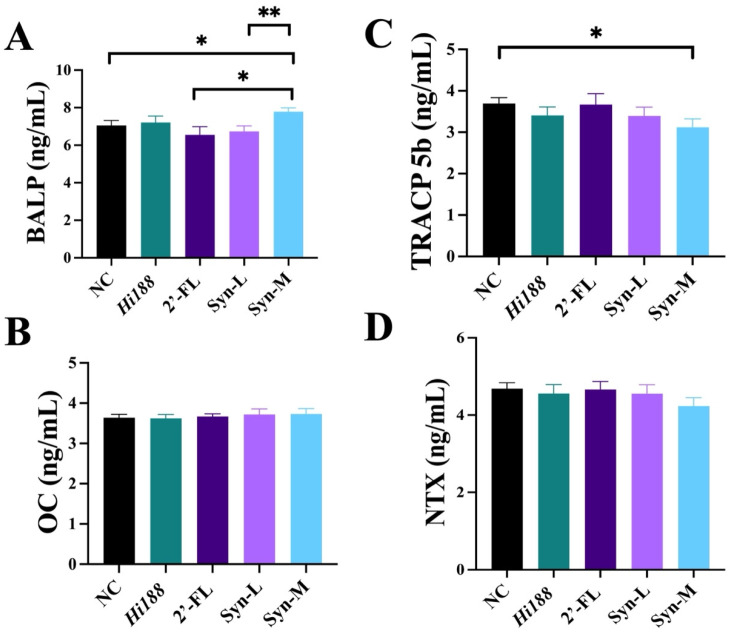
Serum ELISA analyses of different groups. (**A**) Bone-specific alkaline phosphatase (BALP), (**B**) osteocalcin (OC), (**C**) tartrate-resistant acid phosphatase 5b (TRACP-5b), (**D**) Type I collagen *N*-terminal propeptide (NTX). The data are presented as mean ± SEM (*n* = 8). Statistical significance between groups was determined using one-way ANOVA with Tukey’s post hoc test. Different asterisks indicate statistically significant differences among groups (*, *p* < 0.05; **, *p* < 0.01).

To further elucidate the effects of the intervention on bone growth and remodeling, we measured several key bone metabolism-related biochemical markers in serum using an enzyme-linked immunosorbent assay (ELISA), including bone-specific alkaline phosphatase (BALP), osteocalcin (OC), type I collagen *N*-terminal propeptide (NTX), and tartrate-resistant acid phosphatase 5b (TRACP-5b), as shown in Figure 4. As shown in Figure 4A, BALP levels were significantly higher in the Syn-M group compared with the NC group, 2′-FL, and Syn-L groups (*p* < 0.05), showing a 10.39% increase relative to NC. In contrast, serum OC levels showed a slight upward trend across the intervention groups, but no statistically significant differences were observed (*p* > 0.05, Figure 4B). Regarding the bone resorption markers, NTX levels decreased slightly in all intervention groups compared with NC, but these differences were not statistically significant (*p* > 0.05, Figure 4D). Interestingly, TRACP-5b levels were significantly reduced in the Syn-M compared with NC (*p* < 0.05), corresponding to a 15.57% decrease.

### 3.5. Synbiotics Primarily Activated Osteogenesis-Related Pathways

**Figure 5:**
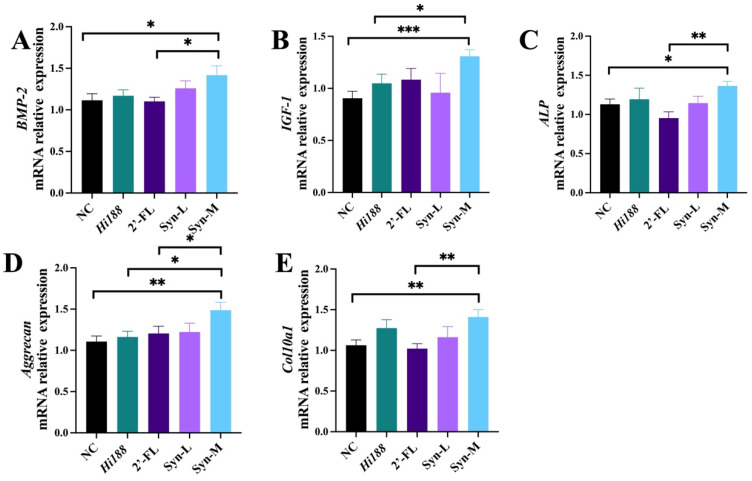
The RT-qPCR results of genes regulating bone growth in mice. (**A**) BMP-2, (**B**) IGF-1, (**C**) ALP, (**D**) Aggrecan, (**E**) Col10a1. Statistical significance was assessed using parametric or non-parametric tests as appropriate, as described in the Section 2.11. Different asterisks indicate statistically significant differences among groups (*, *p* < 0.05; **, *p* < 0.01; ***, *p* < 0.001, *n* = 8).

To further investigate the molecular mechanisms associated with the intervention, the mRNA expression levels related to osteogenesis and extracellular matrix formation were assessed via real-time quantitative PCR (RT-qPCR), including bone morphogenetic protein-2 (BMP-2), insulin-like growth factor-1 (IGF-1), alkaline phosphatase (ALP), aggrecan (AGGRECAN), and collagen type I alpha 1 (Col10a1). Figure 5 shows that BMP-2 expression was significantly increased in the Syn-M group compared with the NC group (*p* < 0.05). Similarly, IGF-1 mRNA levels were markedly increased in Syn-M relative to the NC (*p* < 0.001) and *Hi188* groups (*p* < 0.05) (Figure 5B). ALP expression was also significantly higher in the Syn-M group than in NC (*p* < 0.05) and 2′-FL (*p* < 0.01) (Figure 5C). With respect to extracellular matrix-related genes, aggrecan expression was significantly increased in Syn-M compared with NC (*p* < 0.01), *Hi188* (*p* < 0.05) and 2′-FL (*p* < 0.05) (Figure 5D). In addition, Col10a1 expression was significantly elevated in the Syn-M group relative to NC and 2′-FL (*p* < 0.01) (Figure 5E).

### 3.6. Synbiotics Profoundly Enhance SCFA Production

**Figure 6:**
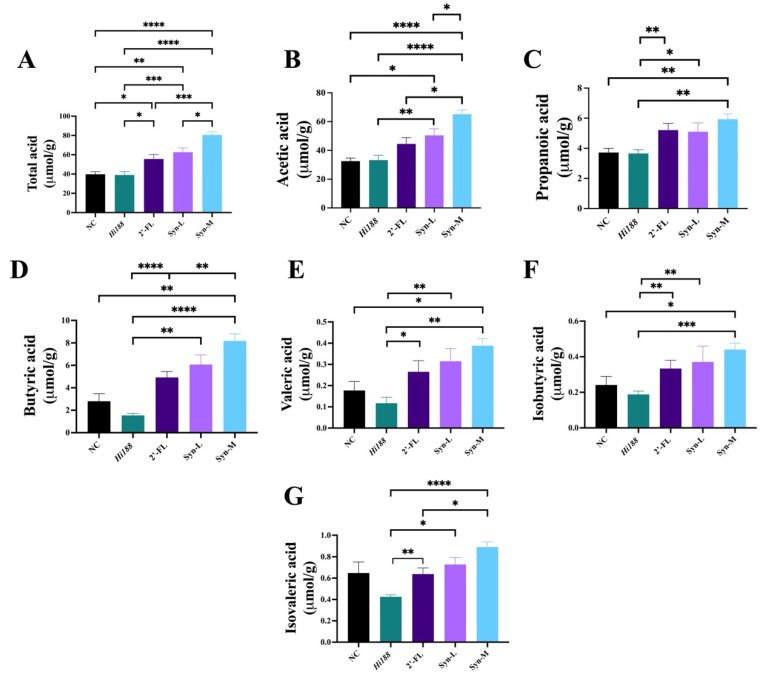
The concentration of different SCFAs detected in mice fecal samples. (**A**) Total acid, (**B**) acetic acid, (**C**) propanoic acid, (**D**) butyric acid, (**E**) valeric acid, (**F**) isobutyric acid, (**G**) isovaleric acid. Statistical significance was assessed using parametric or non-parametric tests as appropriate, as described in the Section 2.11. Different asterisks indicate statistically significant differences among groups (*, *p* < 0.05; **, *p* < 0.01; ***, *p* < 0.001; ****, *p* < 0.0001).

To further investigate the potential relation between gut microbiota metabolism and bone growth, the concentration of short-chain fatty acids (SCFAs), including acetate, propionate, butyrate, valerate, isobutyrate, and isovalerate, was determined in fresh fecal samples (Figure 6). As shown in Figure 6A, the total SCFA production was significantly increased in the Syn-M group compared with the NC, *Hi188*, 2′-FL and Syn-L groups (*p* < 0.05). Both the Syn-L and 2′-FL groups also exhibited higher total SCFA levels than NC. Moreover, Syn-L and Syn-M both showed higher concentrations of total SCFAs than *Hi188* group. Analysis of individual SCFAs revealed a consistent elevation of acetate, propionate, butyrate and valerate in the Syn-M group relative to NC and *Hi188* (*p* < 0.05) (Figure 6B–D). Acetate levels were also significantly higher in Syn-M compared with 2′-FL and Syn-L, while Syn-L showed increased acetate production compared with NC and *Hi188*. For propionate, Syn-L and 2′-FL were significantly higher than *Hi188*, whereas differences between 2′-FL and Syn-L did not reach statistical significance. For butyrate, 2′-FL, Syn-L and Syn-M were significantly higher than *Hi188*, while Syn-M showed increased butyrate production compared with 2′-FL.

With respect to branched-chain and longer-chain fatty acids, valerate and isobutyrate levels were markedly elevated in the Syn-M group compared with NC and *Hi188* (*p* < 0.05) (Figure 6E–G). The 2′-FL and Syn-L groups exhibited intermediate levels, with several SCFAs showing significant increases relative to *Hi188* but remaining lower than those observed in Syn-M.

### 3.7. Synbiotics Improved Gut Metabolic Homeostasis

**Figure 7:**
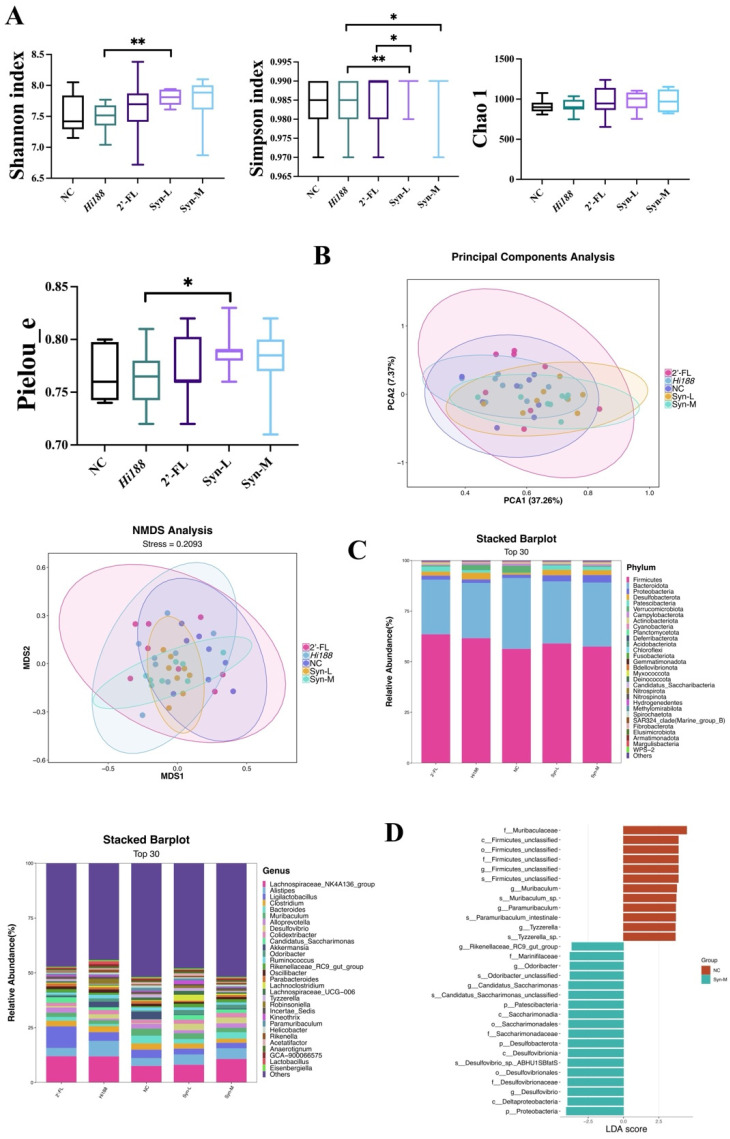
Impact of synbiotic administration on (**A**) alpha diversity (Chao1, pielou_e, Simpson and Shannon indices); (**B**) beta diversity (by non-metric analysis of microbial structure multidimensional scales), and (**C**) bacterial community (phyla, genus); (**D**) cladogram of LDA values of biomarkers in the linear discriminant analysis effect size (LEfSe) analysis. In the cladogram, p, phylum; c, class; o, order; f, family; g, genus; s, species. Data are expressed as means ± SEM (*n* = 8). Different asterisks indicate statistically significant differences among groups. *, *p* < 0.05; **, *p* < 0.01.

To further investigate the microbiota–host interaction mechanisms underlying the effects of *Hi188* and 2′-FL on bone growth in mice, we performed 16S rDNA sequencing on cecal contents. 16S rDNA sequencing is a high-throughput approach that enables comprehensive analysis of the composition and diversity of gut microbiota, thereby revealing alterations in microbial communities. As shown in Figure 7A, α diversity indices, including the Shannon, Simpson and Chao1 indices and Pielou’s evenness (pielou_e), were calculated to evaluate the richness and evenness of the gut microbiota across different groups. Overall, no significant differences were observed between the NC group and the treatment groups (*Hi188*, 2′-FL, low-dose, and medium-dose) across the Shannon, Simpson, Chao1 and pielou_e indices (all *p* > 0.05). However, pairwise comparisons revealed several notable differences among treatment groups. Specifically, the Shannon index in the Syn-L group was significantly higher than that in the *Hi188* group (*p* < 0.05). In the Simpson index, the Syn-L exhibited significant differences relative to both the *Hi188* and 2′-FL groups (*p* < 0.05), and the Syn-M also differed significantly from the *Hi188* group (*p* < 0.05). Furthermore, the Pielou_e index revealed that the Syn-L displayed significantly higher evenness compared with the *Hi188* group (*p* < 0.05). These findings suggest that while the overall diversity and richness were largely unaffected, specific treatment groups exhibited subtle shifts in microbial community evenness and structure.

β-diversity analysis of gut microbiota was evaluated using PCA and NMDS, as shown in Figure 7B. Principal component 1 (PC1) and principal component 2 (PC2) explained 37.26% and 7.37% of the total variance, respectively. The Syn-M group showed a tendency to shift along PC1 compared with NC and single-intervention groups, whereas Syn-L occupied an intermediate position. Consistent patterns were observed in the NMDS ordination based on Bray–Curtis dissimilarity. The Syn-L group clustered closer to the central region, whereas the Syn-M group showed a subtle shift away from the NC cluster, partially overlapping with but distinguishable from the mono-supplemented groups. Permutational multivariate analysis of variance (PERMANOVA) based on Bray–Curtis dissimilarities further revealed a significant overall difference in microbial community composition among experimental groups (PERMANOVA: F = 2.010, R^2^ = 0.186, *p* = 0.001, Supplementary Table S1). These results indicate that dietary interventions significantly contributed to variation in gut microbial structure, despite the absence of complete clustering separation.

Figure 7C illustrates the relative abundance at the phylum and genus levels. At the phylum level, the dominant taxa across all groups were *Firmicutes* and *Bacteroidota*, followed by *Proteobacteria*, *Desulfobacterota*, *Patescibacteria* and *Verrucomicrobiota*. Meanwhile, to further investigate the compositional shifts in gut microbiota at the phylum level, we calculated the ratio of *Firmicutes* to *Bacteroidetes* (F/B ratio) across different intervention groups. No statistically significant differences were observed among any of the groups (all *p* > 0.05, Supplementary Figure S1). At the genus level, the top 30 genera are shown in Figure 7C. Among these, *Lachnospiraceae_NK4A136_group*, *Alistipes*, *Ligilactobacillus*, *Clostridium*, *Bacteroides*, *Muribaculum*, were the most abundant. Compared with the NC group, the relative abundance of *Candidatus_Saccharimonas* increased in the *Hi188*, 2′-FL, Syn-L, and Syn-M groups. whereas *Akkermansia* decreased slightly in Syn-L, and Syn-M groups. *Odoribacter* and *Rikenellaceae_RC9_gut_group* showed higher relative abundance in Syn-L, and Syn-M groups compared that in the NC group. Interestingly, *Desulfovibrio* was enriched in all groups except for NC group.

Furthermore, linear discriminant analysis effect size (LEfSe) was performed to identify taxa differentially enriched between groups (LDA score > 2.0, *p* < 0.05) (Figure 7D). In the NC group, several unclassified taxa within the Firmicutes phylum were significantly enriched, including *f__Muribaculaceae*, *g__Muribaculum*, *g__Paramuribaculum*, and *s__Tyzzerella_*sp., indicating a gut microbiota profile favoring carbohydrate fermentation. Conversely, the Syn-M group was characterized by the enrichment of several metabolically active taxa, such as sulfate-reducing bacteria (*p__Desulfobacterota*, *g__Desulfovibrio*), short-chain fatty acid (SCFA)-producing bacteria (*g__Odoribacter*), *Rikenellaceae_RC9_gut_group*, and *Candidatus_Saccharimonas*. Additionally, members of *Patescibacteria* and *Proteobacteria* were also enriched in Syn-M. These findings suggest that Syn-M intervention promotes the proliferation of functional microbial communities involved in SCFA production and sulfur metabolism, potentially contributing to improved gut metabolic homeostasis.

### 3.8. Spearman Correlations Between Gut Microbiota, SCFAs, and Bone-Related Parameters

**Figure 8:**
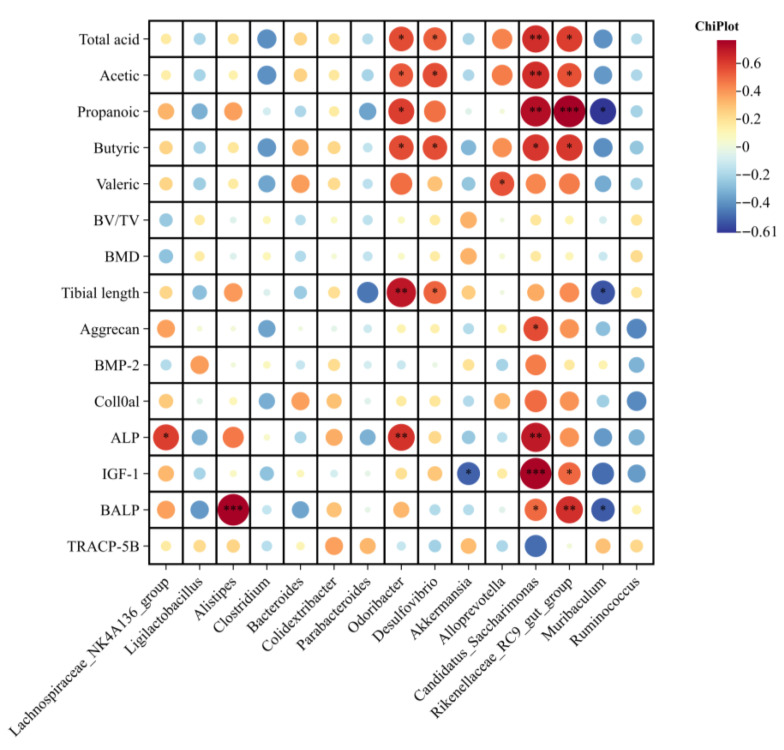
Spearman correlation analysis between dominant gut genera, SCFAs, and bone-related parameters in NC and Syn-M groups. Correlation analysis was performed between gut microbial genera, fecal SCFAs, bone phenotypes, and osteogenic markers using data from NC and Syn-M groups, representing conditions with minimal and maximal biological responses, respectively. *, *p* < 0.05; **, *p* < 0.01; ***, *p* < 0.001. The size of the circle represents the absolute value of the Spearman correlation coefficient. However, no associations remained significant after Benjamini–Hochberg false discovery rate (BH-FDR) correction at FDR < 0.05, and the displayed correlations should therefore be interpreted as exploratory.

Spearman correlation analysis was conducted to assess associations between gut microbial genera, SCFAs, bone phenotypes, and osteogenic markers using three grouping strategies: (i) NC and Syn-M, (ii) NC, Syn-L, and Syn-M, (iii) all experimental groups combined (Figure 8, Supplementary Figures S2 and S3).

At the nominal level, several microbial taxa showed correlations with selected host parameters, particularly SCFA-related variables and a limited number of bone-related indices. However, after Benjamini–Hochberg false discovery rate (BH-FDR) correction for multiple testing, no correlations remained significant at FDR < 0.05 in any of the three analyses. In the restricted analysis comparing the NC and Syn-M groups (Figure 8), the strongest nominal associations were observed for propanoic acid with *Rikenellaceae_RC9_gut_group* and *Candidatus_Saccharimonas*, BALP with *Alistipes*, and IGF-1 with *Candidatus_Saccharimonas*. Nevertheless, none of these correlations remained significant after BH-FDR correction. When the Syn-L group was included (Supplementary Figure S2), the overall nominal correlation patterns were further attenuated, and no association remained significant after multiple-testing correction. Similarly, when all experimental groups were analyzed together (Supplementary Figure S3), the nominal associations were further weakened, and no stable significant correlations were retained after BH-FDR correction.

## 4. Discussion

Bone growth during early life is a highly coordinated biological process involving skeletal cells, systemic endocrine signals, and gut-derived microbial cues. Increasing evidence supports the existence of a functional gut–bone axis, through which intestinal microbiota and their metabolites influence bone modeling, mineral homeostasis, and skeletal development [25,26]. In this study, synbiotic supplementation with 2′-FL and *Hi188*, particularly at a medium dose, was associated with improvements in skeletal growth and bone quality accompanied by structural, biochemical, microbial, and metabolic alterations. These findings suggest that synbiotic intervention may influence skeletal development in association with gut microbiota-related changes during growth.

In the present study, mice were enrolled at 3 weeks of age and fed for 49 days, a period that broadly corresponds to the rapid growth phase spanning juvenile to adolescent development in mice, which is often considered analogous to human childhood and adolescence in terms of skeletal growth dynamics [27,28]. Regarding the whole-body level, synbiotic supplementation did not significantly alter body weight, indicating that the observed skeletal benefits were not attributable to generalized growth acceleration or increased energy intake (Figure 1A,B). Despite comparable body weights across groups, the Syn-M group exhibited a significant increase in tibial length and spine length (Figure 1H,I), suggesting enhanced longitudinal bone growth [29]. Linear bone growth is primarily driven by growth plate activity and endochondral ossification rather than overall body mass [30,31], and thus these results are consistent with a possible selective effect on skeletal elongation during development. In addition to these growth-related changes, organ index analyses showed increased liver and heart indices in the Syn-M group (Figure 1C,D), whereas no significant adverse changes were observed in lung, kidney, or spleen indices (Figure 1E–G). However, because histopathological analyses of the liver and heart were not performed in the present study, the biological significance of the increased liver and heart indices remains uncertain. Colonic histological examination revealed intact mucosal organization and crypt architecture across all groups, with no evidence of epithelial disruption or inflammation (Figure 3). Likewise, preservation of intestinal epithelial integrity and crypt structure suggests no obvious intestinal injury or marked tissue remodeling under the present conditions of synbiotic supplementation [32]. Together, these data do not support gross intestinal structural remodeling as the main explanation for the skeletal phenotype and are more compatible with functional metabolic or signaling-related changes, although these were not directly measured [33].

Consistent with the observed increases in bone length, synbiotic supplementation was associated with improved trabecular bone architecture and mechanical competence. Micro-CT analyses revealed significant increases in BMD, BV/TV, Tb.Th, and Tb.N, accompanied by reduced Tb.sp, in the Syn-M group (Figure 2). These parameters are widely recognized as robust indicators of trabecular structural integrity and bone quality in rodent models [34]. These structural improvements were accompanied by enhanced biomechanical performance, as reflected by increased bending stress and flexural stiffness. The close association between trabecular microarchitecture and mechanical competence has been demonstrated. HR-pQCT analyses showed that they predict biomechanical competence and fracture resistance beyond conventional BMD measures [35]. Such concordance between trabecular reinforcement and mechanical integrity is consistent with physiologically meaningful bone accrual during growth [36].

Biochemical and molecular analyses were broadly consistent with these structural findings. BALP is a well-established marker of osteoblastic activity, and TRACP-5b reflects osteoclast-mediated bone resorption [37,38]. Elevated circulating BALP levels, together with reduced TRACP-5b, are consistent with a bone formation-dominant remodeling state rather than global suppression of bone resorption (Figure 4). At the transcriptional level, the coordinated upregulation of osteogenic regulators (BMP-2, IGF-1, ALP) is compatible with enhanced osteoblast differentiation and matrix mineralization [29,39]. Meanwhile, increased expression of Aggrecan and Col10a1 may reflect altered extracellular matrix turnover and hypertrophic chondrocyte activity within the growth plate (Figure 5) [31,40]. This pattern is broadly consistent with enhanced endochondral ossification, a key process underlying longitudinal bone growth during early life [41]. Importantly, the current changes in osteogenic and chondrogenic markers appear more compatible with coordinated skeletal development than isolated cartilage expansion or uncoupled bone formation.

Although no overt histological alterations were detected in colonic tissues following *Hi188*, 2′-FL, or synbiotic supplementation, this observation does not preclude functional modulation of the gut–bone axis. Microbial metabolic activity and host–microbiota signaling can be substantially altered in the absence of gross morphological changes in intestinal architecture [42]. Shifts in microbial composition and fermentation profiles, including the production of short-chain fatty acids (SCFAs), may occur without detectable epithelial damage or crypt remodeling [43,44,45], especially under physiological dietary interventions [46] rather than inflammatory insults. Moreover, colonic H&E staining primarily reflects structural integrity and inflammatory status but does not capture dynamic alterations in epithelial transport capacity, microbial metabolite flux, or host signaling pathways relevant to mineral metabolism and bone remodeling [47]. Therefore, the absence of apparent histological differences does not exclude the possibility of gut-related functional alterations; however, these observations are insufficient to establish the mechanism underlying the skeletal phenotype.

Measurement of SCFAs in fresh fecal samples demonstrated that synbiotic supplementation robustly enhanced the fecal levels of total SCFAs and individual SCFA species, including acetate, propionate, butyrate, valerate, and branched-chain fatty acids (Figure 6). SCFAs are central microbial metabolites with established roles in mineral absorption, immune modulation, and regulation of osteoclast–osteoblast balance [3,5,18,32,33,34]. Propionate and butyrate have been shown to suppress osteoclast differentiation and bone resorption, thereby shifting bone remodeling toward a formation-dominant state [3,5]. Butyrate has been particularly reported to enhance osteoblast activity through immune-mediated and metabolic mechanisms [18]. The higher fecal SCFA levels observed in the Syn-M group are compatible with the possibility that altered microbial metabolic output was associated with the observed skeletal phenotype.

Microbiota profiling suggested that synbiotic supplementation was associated with remodeling of gut microbial structure potentially relevant to the gut–bone axis. Meanwhile, α-diversity (Shannon and Pielou indices) increased in the synbiotic group, suggesting enhanced ecological evenness. Although β-diversity analyses revealed partial overlap among groups, PERMANOVA supported an overall difference in microbial community structure, and LEfSe analyses identified distinct microbial signatures in the Syn-M groups compared with the NC group, including *Odoribacter*, *Rikenellaceae_RC9_gut_group*, and *Candidatus_Saccharimonas* (Figure 7). However, these taxa should be interpreted cautiously, as LEfSe identifies differentially enriched microorganisms but does not establish functional relevance or causal links to host skeletal phenotypes.

To further explore potential microbiota–host relationships, we performed Spearman correlation analysis between selected bacterial genera, fecal SCFAs, and bone-related parameters. In the restricted-group analysis, *Odoribacter*, *Rikenellaceae_RC9_gut_group*, and *Candidatus_Saccharimonas* showed normal positive associations with selected fecal SCFAs and some skeletal or osteogenic indices, whereas *Muribaculum* showed normal negative associations with several variables. These findings are compatible with the possibility that synbiotic supplementation was accompanied by parallel changes in microbial composition, fecal metabolites, and bone-related outcomes. However, these correlations should be interpreted with caution and regarded as exploratory and hypothesis-generating only, because most signals were attenuated in the broader-group analysis and none remained significant after BH-FDR correction. Among the taxa enriched in the Syn-M group, *Odoribacter* has been reported in some contexts as an SCFA-associated genus, particularly with respect to butyrate production, and has been discussed in relation to host metabolic regulation [48]. *Rikenellaceae_RC9_gut_group* also showed nominal positive correlations with SCFAs, IGF-1 expression and serum BALP levels in the unadjusted analysis, although these associations were not retained after BH-FDR correction. Therefore, the present data do not provide robust statistical support for a specific link between this taxon and bone-related outcomes. Previous studies have shown that this genus can participate in host energy metabolism by producing SCFAs, such as acetic acid and propionic acid [49]. Moreover, Mendelian randomization analysis suggests that *Rikenellaceae* may have a genetically protective association with bone mineral density [50]. Accordingly, this taxon may still be considered a candidate for further investigation, rather than a confirmed mediator in the present study. In addition, *Candidatus_Saccharimonas*, a member of the *Saccharibacteria* (TM7) phylum, also showed nominal positive associations with bone growth indices and osteogenic markers before BH-FDR. Recent studies suggest that TM7-related bacteria may participate in host immune regulation, and, in specific contexts, may indirectly relate to bone homeostasis. In the oral cavity, TM7 epibionts have been shown to suppress inflammatory bone loss by modulating host bacterial pathogenicity [51]. The increase in *Desulfovibrio* may reflect an additional microbial shift associated with synbiotic supplementation. Previous studies have linked *Desulfovibrio* to sulfur metabolism and redox-related intestinal processes [52]; however, the functional significance of this change was not directly evaluated in the present study. The microbiome findings suggest that medium-dose synbiotic supplementation was associated with modest but detectable shifts in gut microbial composition and fecal metabolic profiles alongside improved bone-related phenotypes.

Several limitations of the present study should be acknowledged. First, SCFAs were measured in freshly voided fecal samples, which reflect residual microbial metabolic output rather than intestinal absorption, circulating exposure, or bone tissue receptor signaling; accordingly, the link between fecal SCFAs and skeletal outcomes should be interpreted as correlative rather than causal. Second, the correlation analyses performed in this study were exploratory in nature. After correction for multiple comparisons using the Benjamini–Hochberg false discovery rate procedure, no association remained significant at FDR < 0.05, indicating that the potential links among gut microbial taxa, fecal SCFAs, and bone-related parameters require further validation in independent and more targeted studies. Third, although transcriptional and biochemical markers provide molecular support for altered bone remodeling, future studies incorporating cell-specific histomorphometric analyses or isotope-based mineral flux measurements would further refine the understanding of cellular contributions to the observed skeletal phenotypes. Fourth, femur length was not measured. Although the support span was kept constant across all groups during the three-point bending test, this parameter may be relevant for a more comprehensive interpretation of the biomechanical findings. Fifth, only one dose level was included for *Hi188* alone and 2′-FL alone, whereas two reduced-dose combination groups were tested for the synbiotic intervention. As a result, the current design does not permit direct dose-matched comparisons and is insufficient to rigorously define dose–response relationships or statistically confirm synergistic effects. Sixth, mice were group-housed, which may have introduced a cage effect in the gut microbiota analysis, as co-housed animals can exchange microorganisms through coprophagy; therefore, the 16S rRNA sequencing results should be interpreted with this consideration in mind. Seventh, histopathological analyses of the liver and heart were not performed, and thus the biological significance of the increased liver and heart indices observed in the Syn-M group remains unclear. Finally, although the present study integrates phenotypic, microbial, metabolic, and molecular observations, it does not establish the intracellular signaling pathways that may underlie the association between synbiotic supplementation and improved bone-related outcomes. These mechanisms were not directly investigated and remain to be clarified in future targeted studies.

Despite these limitations, the convergence of structural, mechanical, molecular, microbial, and metabolic findings provides a broadly coherent pattern suggesting that synbiotic supplementation was associated with altered gut microbial and metabolic profiles, alongside improved skeletal development.

## 5. Conclusions

The present study shows that synbiotic supplementation with 2′-fucosyllactose and *Lactiplantibacillus plantarum Hi188*, particularly at the medium dose, was associated with improved skeletal growth and bone quality during early development. These effects were reflected by enhanced longitudinal bone growth, improved trabecular microarchitecture and mechanical properties, coordinated changes in bone turnover markers, and upregulation of osteogenesis- and matrix-related genes. Notably, these changes occurred without marked alterations in generalized body growth or intestinal morphology and were accompanied by altered fecal SCFA profiles and gut microbial composition. Taken together, these findings support the biological plausibility of a gut microbiota–bone association during development, and suggest that targeted synbiotic supplementation may represent a promising nutritional strategy for supporting skeletal health in early life.

## Figures and Tables

**Table 1:** Primer sequences of bone-related genes used for qRT-PCR.

Gene	Organism	Sequence 5′ to 3′	Annealing T °C
GAPDH	Mouse	F: TGACCTCAACTACATGGTCT	60
		R:CTTCCCATTCTCGGCCTTG	
AGGRECAN	Mouse	F: CAGTGCGATGCAGGCTGGCT	60
		R: CCTCCGGCACTCGTTGGCTG	
ALP	Mouse	F: GCTTTAAACCCAGACACAAG	59
		R: AAGAAGAAGCCTTTGAGGTT	
BMP-2	Mouse	F: AAGAGACATGTGAGGATTAGCAGGT	60
		R: GCTTCCGCTGTTTGTGTTTG	
COL10A1	Mouse	F: TTCCGAGTATGACT	60
		R: GCCAATATCAGTCGGGAACA	
IGF-1	Mouse	F: ACAGGCTATGGCTCCAGCATTC	60
		R: GCACAGTACATCTCCAGTCTCCTC	

## Data Availability

The datasets generated for this study can be found in the NCBI SRA repository under the accession number PRJNA1404667; https://www.ncbi.nlm.nih.gov (accessed on 25 March 2026).

## Supplementary Materials

The following supporting information can be downloaded at: https://www.mdpi.com/article/10.3390/nu18071123/s1, Figure S1: Firmicutes-to-Bacteroidota ratio across experimental groups. No statistically significant differences were observed among groups (mean ± SD, *p* > 0.05); Figure S2: Spearman correlation analysis between gut microbiota, SCFAs, and bone-related parameters in NC and synbiotic-treated groups. Spearman correlation analysis was performed to assess associations between the relative abundance of selected bacterial genera and host parameters, including fecal short-chain fatty acids (SCFAs) and bone-related indices (BV/TV, BMD, tibial length, and osteogenic markers). The analysis was restricted to the NC, Syn-L, and Syn-M groups. Correlation coefficients are represented by color intensity (red, positive correlation; blue, negative correlation), and circle size reflects the magnitude of the correlation coefficient. The size of the circle represents the absolute value of the Spearman correlation coefficient. Asterisks indicate statistically significant correlations (*, *p* < 0.05; **, *p* < 0.01). However, no associations remained significant after Benjamini–Hochberg false discovery rate (BH-FDR) correction at FDR < 0.05, and the displayed correlations should therefore be interpreted as exploratory; Figure S3: Spearman correlation analysis between gut microbiota, SCFAs, and bone-related parameters across all experimental groups. Spearman correlation analysis was conducted using data from all experimental groups (NC, 2’-FL, *Hi188*, Syn-L, and Syn-M) to evaluate associations between bacterial genera and metabolic or skeletal parameters. Correlation coefficients are indicated by color gradient (red, positive; blue, negative), with circle size proportional to correlation strength. The size of the circle represents the absolute value of the Spearman correlation coefficient. Statistical significance is denoted by asterisks (*, *p* < 0.05; **, *p* < 0.01). However, no associations remained significant after Benjamini–Hochberg false discovery rate (BH-FDR) correction at FDR < 0.05, and the displayed correlations should therefore be interpreted as exploratory; Figure S4: Representative scheme of the animal study design described in Section 2.2, including mouse grouping, dietary intervention, gavage treatment, and sample collection; Table S1: PERMANOVA results for β-diversity analysis based on Bray–Curtis distances.
