# Supplementary material for: Effect of a Synbiotic Combination of 2′-Fucosyllactose and Lactiplantibacillus plantarum Hi188 on Skeletal Growth and Gut Microbial Metabolism in Growing Mice
Source: Nutrients. 2026 Mar 31;18(7):1123. doi: 10.3390/nu18071123 (PMC13074401; doi:10.3390/nu18071123)
Supplement: Supplementary file 1 [file nutrients-18-01123-s001.zip › nutrients-4207125-supplementary materials.pdf]

Table S1 PERMANOVA results for β-diversity analysis based on Bray–Curtis distances

	Df	SumOfSqs	R2	F	Pr(>F)
Group	4	1.564	0.186	2.010	0.001
Residual	35	6.826	0.814		
Total	39	8.39	1		

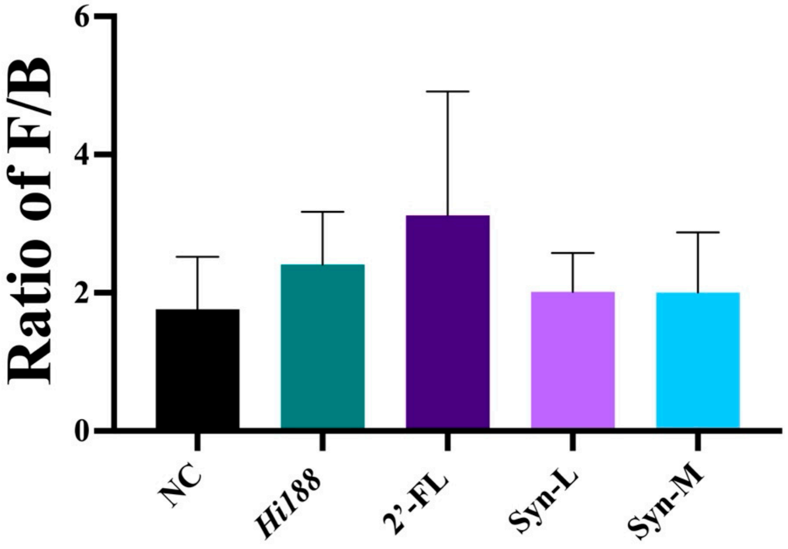

Figure S1. *Firmicutes*-to-*Bacteroidota* ratio across experimental groups. No statistically significant differences were observed among groups (mean ± SD, $p > 0.05$).

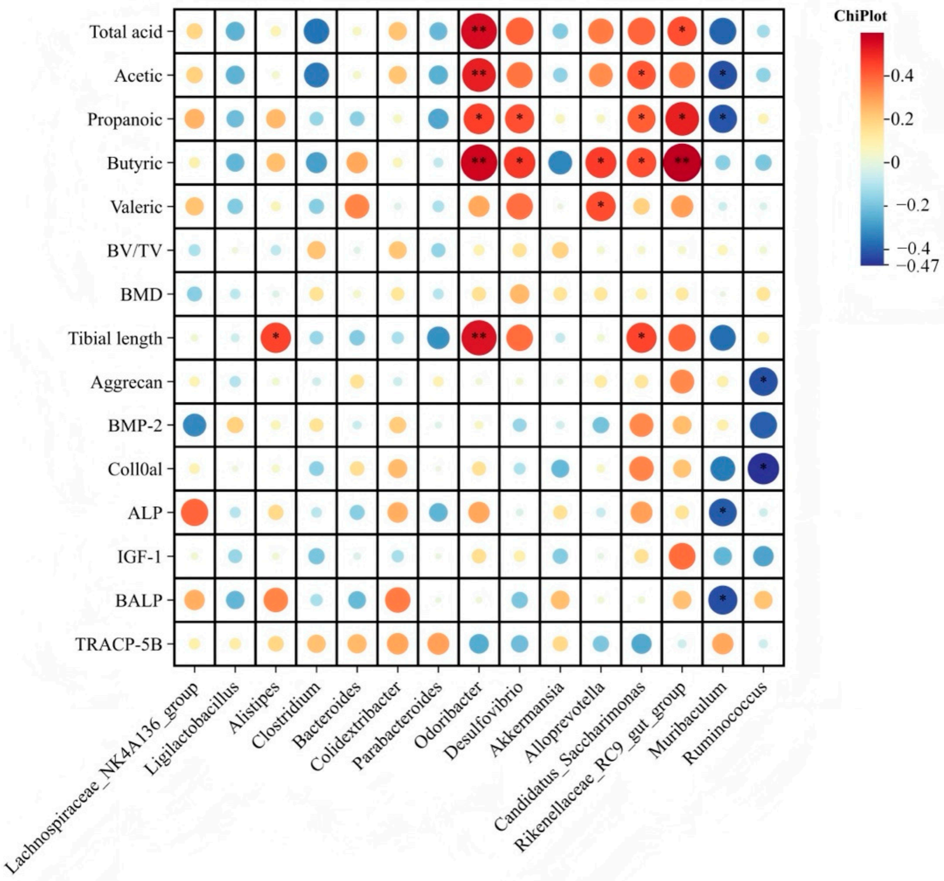

Figure S2. Spearman correlation analysis between gut microbiota, SCFAs, and bone-related parameters in NC and synbiotic-treated groups. Spearman correlation analysis was performed to assess associations between the relative abundance of selected bacterial genera and host parameters, including fecal short-chain fatty acids (SCFAs) and bone-related indices (BV/TV, BMD, tibial length, and osteogenic markers). The analysis was restricted to the NC, Syn-L, and Syn-M groups. Correlation coefficients are represented by color intensity (red, positive correlation; blue, negative correlation), and circle size reflects the magnitude of the correlation coefficient. The size of the circle represents the absolute value of the Spearman correlation coefficient. Asterisks indicate statistically significant correlations (*,$p < 0.05$; **,$p < 0.01$). However, no associations remained significant after Benjamini–Hochberg false discovery rate (BH-FDR) correction at FDR < 0.05, and the displayed correlations should therefore be interpreted as exploratory.

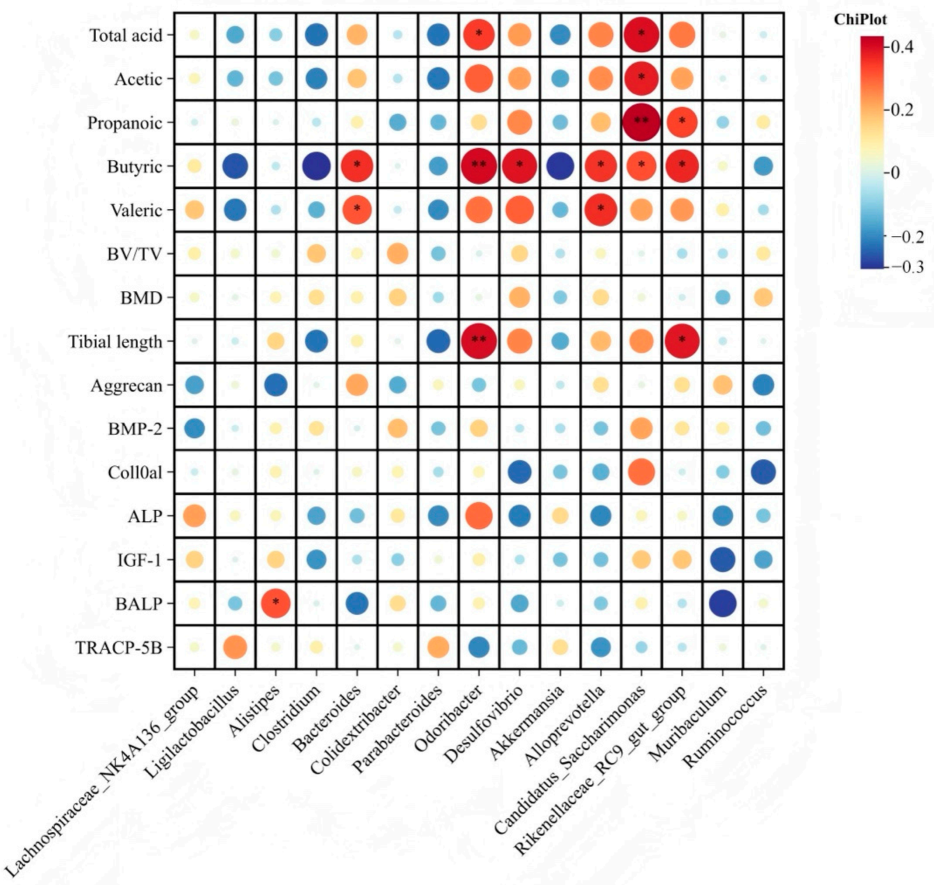

Figure S3. Spearman correlation analysis between gut microbiota, SCFAs, and bone-related parameters across all experimental groups. Spearman correlation analysis was conducted using data from all experimental groups (NC, 2’-FL, *Hi188*, Syn-L, and Syn-M) to evaluate associations between bacterial genera and metabolic or skeletal parameters. Correlation coefficients are indicated by color gradient (red, positive; blue, negative), with circle size proportional to correlation strength. The size of the circle represents the absolute value of the Spearman correlation coefficient. Statistical significance is denoted by asterisks (*,p < 0.05; **,p < 0.01). However, no associations remained significant after Benjamini–Hochberg false discovery rate (BH-FDR) correction at FDR < 0.05, and the displayed correlations should therefore be interpreted as exploratory.

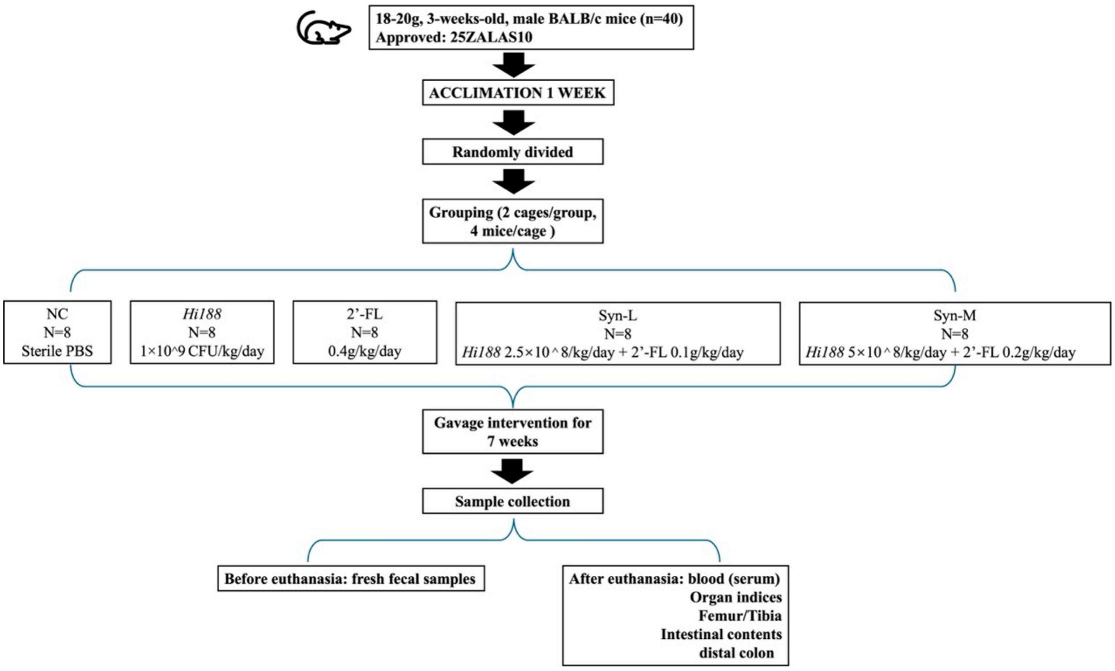

Supplementary Figure S4. Representative scheme of the animal study design described in Section 2.2, including mouse grouping, dietary intervention, gavage treatment, and sample collection.
